# Dr. James Thomas Jelks

**Published:** 1902-08

**Authors:** 


### OBITUARY.

Dr. James Thomas Jelks, of Hot Springs, Arkansas, died suddenly at his home in that city, June 24, 1902, aged 53 years. He was stricken down with an affection of the heart in the midst of the greatest activity of his career as a successful physician, and his death was a shock to a large circle of friends and acquaintances throughout the country.

Dr. Jelks was born in Russell County, Ala., May 20, 1849. He served in the confederate army during the last years of the civil war, at the close of which he devoted his time to the study of medicine and graduated from the University of Nashville, medical department, in 1870. He first practised at Culloden, Ga; from there he went to Marietta, in the same state, and afterward located at St. Louis, Mo. While practising in the latter city the health of one of his family necessitated his removal to Hot Springs, which he did in 1877, where he remained until his death.

In 1883 he was appointed to a professorship of genitourinary surgery and venereal diseases in the College of Physicians and Surgeons at Chicago, which he retained until 1890, and in 1892 was appointed to the chair of gynecology and syphilology in Barnes's Medical College, at St. Louis, holding this place until his death. He was a member of numerous medical societies and published several magazine articles of scientific value. He was a member of the Southern Surgical and Gynecological Associaticn and a Fellow of the American Association of Obstetricians and Gynecologists. He was also one of the editors of the *Hot Springs Medical Journal*. He is survived by a widow and two sons, Dr. F. W. Jelks, of St. Louis, and James T. Jelks, Jr., of Hot Springs.

